# Magnitude, Presentation, and Management of Pelvic Organ Prolapse at a Tertiary Referral Hospital in Southwestern Uganda: Retrospective Medical Records Review (2014–2018)

**DOI:** 10.7759/cureus.102295

**Published:** 2026-01-26

**Authors:** Musa Kayondo, Rogers Kajabwangu, Dan Kaye, Richard Migisha, Brenda Ainomugisha, Onesmus Byamukama, Kalyebara Paul Kato, Henry M Lugobe, Verena Geissbüehler

**Affiliations:** 1 Obstetrics and Gynaecology, Mbarara University of Science and Technology, Mbarara, UGA; 2 Obstetrics and Gynaecology, Makerere University, Kampala, UGA; 3 Physiology, Mbarara University of Science and Technology, Mbarara, UGA; 4 Obstetrics and Gynaecology, University of Basel, Geneva, CHE

**Keywords:** magnitude, management, pelvic organ prolapse, presentation, uganda

## Abstract

Purpose: This study sought to determine the burden, clinical characteristics, and management approaches of pelvic organ prolapse (POP) among women admitted to the gynecology unit at Mbarara Regional Referral Hospital (MRRH) in southwestern Uganda.

Methods: A retrospective review was conducted of medical records for women treated for pelvic floor disorders (PFDs) at the gynecology unit of MRRH between January 2014 and December 2018. The PFDs evaluated included POP, genitourinary fistulas, anosphincter injuries, and other forms of urinary incontinence. For women diagnosed with POP, additional information regarding the type, stage, treatment modality, and postoperative complications was extracted. Descriptive statistics were summarized using frequencies, and comparisons were made using the chi-square test, with statistical significance set at p < 0.05.

Results: During the study period, 9,109 women were admitted to the gynecology unit, of whom 674 (7.4%) were diagnosed with PFDs. POP accounted for 210 (31.2%) of these cases, yielding a POP prevalence of 2.3% (95% CI: 2.0-2.61%). Uterine prolapse was the predominant type, observed in 77.1% of cases. The majority of patients (86.2%) presented with advanced disease (stage III or IV), and most (94.2%) received surgical treatment. Early postoperative complications occurred in 7.1% (14/198) of surgically managed patients, with vaginal cuff sepsis being the most frequent complication (5.1%; 10/198).

Conclusion: POP represents a significant proportion of gynecological admissions at MRRH and is predominantly treated surgically with a low rate of early postoperative complications. Preventive strategies should be strengthened to reduce the occurrence of POP, and greater emphasis should be placed on promoting less invasive treatment options, including the use of pessaries.

## Introduction

Pelvic organ prolapse (POP) is a condition characterized by the downward displacement of one or more pelvic organs into, or beyond, the vaginal canal due to progressive weakening of the pelvic floor support structures [1]. The organs commonly involved include the bladder, uterus, rectum, and, in some cases, the small intestine [1,2]. Based on the anatomical compartment affected, POP may present as anterior compartment prolapse (cystocele, urethrocele, or both), posterior compartment prolapse (rectocele or enterocele), or apical compartment prolapse, which includes uterine prolapse and vaginal vault prolapse [3]. POP forms part of a broader group of conditions collectively referred to as pelvic floor disorders (PFDs), which also include urinary and fecal incontinence, anosphincter injuries, and obstetric fistula [4]. For clinical evaluation and standardized reporting, POP severity is graded from stage I to stage IV using the Pelvic Organ Prolapse Quantification (POP-Q) system, as validated by the International Continence Society [5,6].

POP is a common condition among aging and parous women worldwide. It is estimated that approximately half of parous women aged 50 years and older experience some degree of POP [7], with lifetime prevalence estimates ranging between 30% and 50% following menopause [8]. In high-income countries, reported prevalence varies considerably, with estimates of about 42% among women aged 50-79 years in the United States [9], 8.3% in Sweden [10], and 8.4% in the United Kingdom [11]. In contrast, data from low-income countries remain limited. Available hospital-based studies report prevalence rates of 1.4% among gynecological admissions in Nigeria [12], 2.68% in Ghana [13], and 23.5% in Ethiopia [14], highlighting substantial regional variation.

POP has been shown to adversely affect women’s quality of life by causing physical discomfort, functional impairment, and psychosocial distress [15]. Management options include both conservative and surgical approaches. Conservative treatment modalities, particularly vaginal pessaries, have demonstrated effectiveness comparable to surgical intervention in appropriately selected patients [16-18]. However, their utilization remains low in many low-resource settings, largely due to limited availability and access [19]. Surgical management, while effective in improving symptoms and quality of life, is associated with potential perioperative complications, including infection, hemorrhage requiring blood transfusion, bowel injury, and urinary tract injury, especially involving the bladder [20]. Previous studies have reported perioperative complication rates ranging from 11.3% to 46% [21-23]. Despite this, information on the burden of POP, patterns of management, and associated complications remains scarce in southwestern Uganda. This study therefore aimed to determine the magnitude of POP among gynecological admissions at Mbarara Regional Referral Hospital (MRRH) and to describe patient characteristics and management practices.

This article was previously presented as a poster at the International Urogynecological Association (IUGA) 49th Annual Meeting, held from June 19-22, 2024, in Singapore.

## Materials and methods

Study setting

This study was carried out at the Gynecology Unit of MRRH, a public tertiary referral facility located in southwestern Uganda. The gynecology department provides specialized tertiary-level care, including urogynecology services, delivered by both resident specialists and visiting surgeons.

Study design

We conducted a retrospective review of medical records for women who were diagnosed and managed for PFDs at the gynecology unit of MRRH over a five-year period, from January 2014 to December 2018.

Study population and enrollment

Medical charts of all women admitted to the Gynecology Unit of MRRH during the study period were reviewed. From this cohort, records with a documented diagnosis of a PFD were included. A patient was classified as having a PFD if the diagnosis included any of the following conditions: POP, anosphincter injury, genitourinary fistula, stress urinary incontinence, urge urinary incontinence, or overflow incontinence. POP was defined by the presence of at least one of the following conditions: cystocele, urethrocele, cystourethrocele, uterine prolapse, vaginal vault prolapse, enterocele, or rectocele.

Data collection and study variables

Data were extracted using a standardized medical record abstraction tool designed for the study. Information collected included participant characteristics, such as age, parity, occupation, and marital status, as well as the specific type of PFD diagnosed (POP, anosphincter injury, stress urinary incontinence, urge incontinence, or overflow incontinence).

For participants diagnosed with POP, additional variables were obtained, including the anatomical type of prolapse, preoperative stage of prolapse (stages I-IV), and the management approach employed (surgical or conservative). Intraoperative data collected comprised the type of surgical procedure performed and the occurrence of complications, including bladder injury, bowel injury, ureteric injury, and intraoperative bleeding requiring blood transfusion. The post-operative data included (1) complications such as infection of the vaginal cuff and (2) time spent on the ward post-operatively in days. Other postoperative outcomes, such as readmission, reoperation, or long-term complications, were not systematically assessed because these were not consistently documented in the retrospective medical records.

Vaginal cuff infection was diagnosed based on the presence of lower abdominal pain, purulent vaginal discharge, and tenderness at the surgical site [24]. POP staging was performed using the POP-Q system [5]. Data abstraction was conducted by trained research assistants.

Statistical analysis

Data were entered into REDCap software and subsequently exported to Stata version 17 (StataCorp, LLP, College Station, TX) for statistical analysis. Categorical variables were summarized using frequency distributions. The magnitude of POP was assessed using two measures: the prevalence of POP among all gynecological admissions and the proportion of POP among women diagnosed with PFDs. Prevalence was calculated by dividing the number of women with POP by the total number of gynecology admissions during the study period and expressing the result as a percentage. The proportion of POP among PFD cases was determined by dividing the number of women with POP by the total number of women diagnosed with any PFD during the same period. Comparisons between women with POP and those with other PFDs were performed using the chi-square test.

Ethical considerations

Ethical approval was obtained from the MRRH Research Ethics Committee (reference: 17/08-18) and the Uganda National Council for Science and Technology (reference: HS368ES). A waiver of informed consent was granted due to the retrospective nature of the study and the use of existing medical records.

## Results

We retrieved a total of 9,109 files of patients admitted to the Gynecology Unit between January 2014 and December 2018. Of these, 684 had a diagnosis of PFD, of which 674 (98.5%) had complete data, and these are the ones that were included in the data extraction and analysis. The participants' characteristics are shown in Table 1. The majority of participants with PFD were aged 18-34 years (42.6%), peasant farmers (91.8%), and married (62%). When compared to other PFDs, participants with POP were significantly older (>60 years) and of higher parity (>5) (Table 1).

**Table 1:** Baseline characteristics of the study participants Values are presented as frequencies and percentages N (%). Pelvic organ prolapse (POP) was compared with other pelvic floor disorders (PFDs) using Pearson's chi-square test. χ² indicates the chi-square statistic, and df denotes degrees of freedom. A p value < 0.05 was considered statistically significant.

Socio-demographic Characteristics (n= 674)		Overall N=674 (%)	Pelvic Organ Prolapse n= 210 (%)	Other Pelvic Floor Disorders n= 464 (%)	χ² (df)	P value
Age	18-34	287 (42.6)	9 (3.1)	278 (96.9)	276.93 (3)	<0.001
	35-49	192 (28.5)	61 (31.8)	131 (68.2)		
	50-59	82 (12.2)	44 (53.7)	38 (46.3)		
	≥ 60	113 (16.7)	96 (85)	17 (15)		
Occupation	Peasant	619 (91.8)	205 (33)	414 (67)	14.20 (2)	0.001
	Business	36 (5.4)	2 (5.6)	34 (94.4)		
	Professional	19 (2.8)	3 (15.8)	16 (84.2)		
Marital Status	Married	418 (62)	91 (21.7)	327 (78.3)	44.06 (1)	<0.001
	Single	256 (38)	119 (46.5)	137 (53.5)		
Parity	0	10 (1.5)	1 (10)	9 (90)	172.88 (2)	< 0.001
	1-4	346 (51.3)	31 (9)	315 (91)		
	≥ 5	318 (47.2)	178 (56)	140 (44)		

Magnitude of POP at MRRH

A total of 9,109 patients were admitted on the gynecology ward, and the prevalence of POP among gynecology admissions at MRRH was 2.3% (95%CI: 2-2.61%).

**Figure 1:**
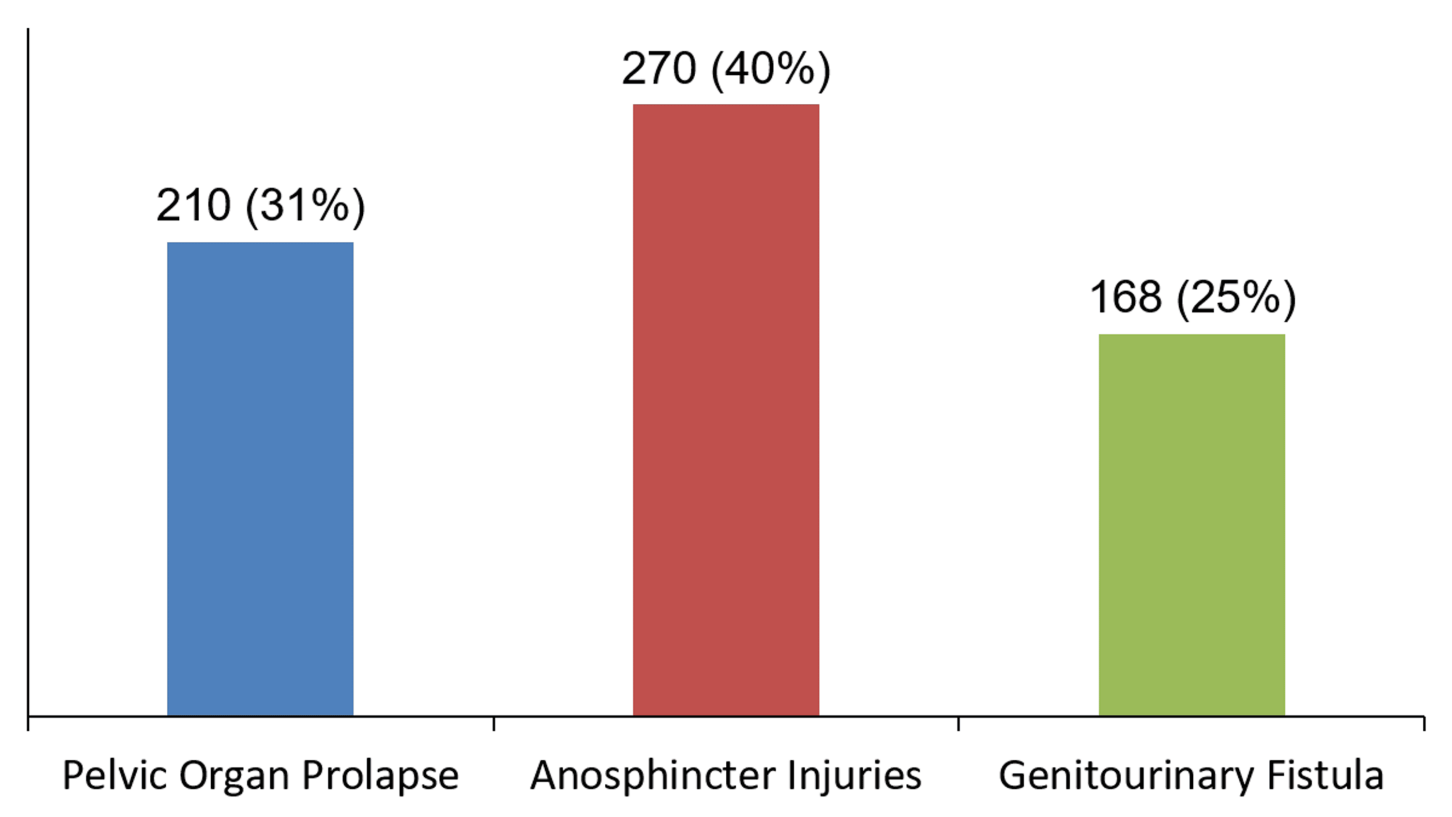
Distribution of pelvic floor disorders among gynecology admissions at Mbarara Regional Referral Hospital (2014–2018) Bars represent the number of cases and corresponding percentages.

Among all cases of PFDs from 2014 to 2018, POP accounted for 31% of cases (95%CI: 27.7-34.8%) compared to 40% for anosphincter injuries and 25% for genitourinary fistula, hence making it the second most common PFD, as shown in Figure 1.

Description of POP by type and stage

The types and stages of POP among the study participants with POP are shown in Table 2. The most common type of POP was uterine prolapse, detected in 77.1% of the cases. The majority (86.2%) presented with high stages of POP (stage III or stage IV). No participant presented with an enterocele.

**Table 2:** Proportion of pelvic organ prolapse by type and stage Data are presented as N (%). POP: pelvic organ prolapse

Variable		Frequency (n)	Percentage (%)
Type of POP (n=210)			
Uterine prolapse	Yes	162	77.1
	No	48	22.9
Cystocele	Yes	134	63.8
	No	76	36.2
Rectocele	Yes	38	18.1
	No	172	81.9
Vault prolapse	Yes	14	6.7
	No	196	93.3
Stage of POP (n=210)			
Stage II		29	13.8
Stage III		92	43.8
Stage IV		89	42.4

Management of POP at MRRH

Among the 210 participants who had POP, 198 (94.3%) were treated surgically, while the rest (12, 5.7%) were managed conservatively with pelvic floor exercises. No patients were managed with pessaries. The different surgeries performed and complications encountered are shown in Table 3. Vaginal hysterectomy with sacrospinous ligament vault fixation was the most common surgery that was performed in 111 (56%) of the participants. The intraoperative complications encountered included rectal injuries (n=2), bleeding requiring blood transfusion (n=1), and injury to the urinary bladder (n=1). Vaginal cuff infection was the most common (5.1%) postoperative complication.

**Table 3:** Surgeries performed in the study participants and related complications Data are presented as N (%).

Surgical procedures n=198	n (%)
Vaginal hysterectomy plus sacrospinous ligament vault fixation	111 (56)
Vaginal hysterectomy with anterior and posterior repair plus uterosacral ligament vault fixation	28 (14.1)
Posterior colporrhaphy alone	15 (7.6)
Anterior colporrhaphy alone	21 (10.6)
Sacrospinous vault fixation for vault prolapse	10 (5.1)
Anterior and Posterior Colporrhaphy	8 (4)
Sacrospinous hysteropexy	5 (2.5)
Complications (n=14)	
Intraoperative	4 (28.5)
Postoperative	10 (71.5)

## Discussion

This retrospective chart review assessed the burden of POP and described the clinical characteristics and management patterns among women admitted to MRRH between 2014 and 2018. The prevalence of POP among gynecology admissions was 2.3%, and POP emerged as the second most common PFD after anosphincter injuries. Women diagnosed with POP were predominantly older and of higher parity compared to those with other PFDs. Uterine prolapse was the most frequently observed type, with most patients presenting at advanced stages (stage III or IV). Surgical management was the predominant treatment approach, and postoperative complications were relatively infrequent.

The observed POP prevalence of 2.3% is comparable to findings reported in other hospital-based studies conducted in sub-Saharan Africa. Similar prevalence rates of 1.4% and 2.68% among gynecological admissions have been reported in Nigeria and Ghana, respectively [12,13]. These similarities may be explained by comparable study designs and populations, as all studies were conducted in tertiary hospital settings among admitted gynecological patients. In addition, shared socio-demographic characteristics, such as low socioeconomic status and high parity, which are well-established risk factors for POP [9,25-28], may further account for the comparable prevalence. In the present study, the predominance of peasant farmers and multiparous women likely contributed to the observed burden.

In contrast, substantially higher prevalence estimates ranging from 20% to 46% have been reported in studies from Ethiopia, Uganda, and Gambia [28-30]. These differences may be attributed to variations in study methodology, particularly case ascertainment and recruitment strategies. Community-based surveys, such as those conducted in Ethiopia and Gambia, often rely on self-reported symptoms without routine pelvic examination, potentially leading to overestimation of POP prevalence. Similarly, a study by Tugume et al. conducted at the gynecology outpatient department of MRRH included both symptomatic and asymptomatic women, which likely contributed to the higher reported prevalence [29]. In contrast, the present study was inpatient-based and included only women with clinically confirmed POP who presented for definitive management.

With respect to the distribution of PFDs, POP accounted for 31.2% of all cases, ranking second after anosphincter injuries, while genitourinary fistula ranked third. This pattern is consistent with findings from studies conducted in Ethiopia and western Uganda, where POP similarly ranked as the second most common PFD [9,30,31]. These similarities likely reflect shared contextual factors, including limited access to skilled obstetric care in many low-resource settings [14,32-35], which increases the burden of delivery-related complications, such as anosphincter injuries and fistulas.

Most women with POP in this study were postmenopausal and had high parity (five or more deliveries), findings that are consistent with previous reports [36,37]. The increased occurrence of POP among postmenopausal women may be related to age-associated weakening of pelvic floor musculature and connective tissue, compounded by declining estrogen levels [38]. High parity likely contributes through cumulative stretching and injury to pelvic support structures during repeated pregnancies and vaginal deliveries [39]. However, evidence from a study in the United States suggests that the degree of pelvic floor injury sustained during vaginal delivery, rather than parity alone, may be a more important determinant of POP risk [40].

Surgical intervention was the primary mode of POP management at MRRH, with 94.2% of patients undergoing operative treatment. This high surgical rate is notable given that conservative management options, particularly vaginal pessaries, have demonstrated effectiveness comparable to surgical treatment [16-18,41]. In this study, POP was predominantly diagnosed at advanced stages, with more than four-fifths of women presenting with stage III or IV disease, while only a small proportion presented with stage II prolapse and none with stage I. Even among women with advanced prolapse, pessaries have been shown to provide outcomes comparable to surgery [17,42,43]. Despite this, no patient in the present review was managed with a pessary. This low utilization likely reflects the limited availability, accessibility, and acceptability of pessaries in this setting. Previous studies have similarly reported that pessaries are often unavailable and that many women prefer surgical correction even after counseling [17,41,44].

The overall postoperative complication rate in this study was 7.1%, with the most common complications being visceral injuries involving the urinary bladder or rectum and postoperative vaginal cuff infection. This rate is comparable to complication rates reported in other studies, which range from 2.5% to 11.3% [17,22,45,46]. The low complication rate could be due to the short follow-up period, as postoperative outcomes were assessed only during the inpatient stay, typically up to one week after surgery. Readmission, reoperation, and longer-term postoperative complications were not captured in this review, which may have led to an underestimation of the true postoperative complication rate. Studies with longer follow-up durations have reported higher complication rates, ranging from 10% to 25% [47], suggesting that delayed complications may not have been fully captured in the present review.

Limitations

This study was conducted at a single tertiary referral center and included only women admitted with gynecologic disorders, which may limit the generalizability of the findings. The prevalence reported is therefore a hospital-based prevalence. Future multicenter and community-based studies are recommended to better characterize the prevalence and burden of POP across diverse populations in Uganda. Additionally, information on the duration and complexity of surgical procedures was not consistently documented in the medical records. As a result, it was not possible to determine whether the observed vaginal cuff infections were associated with operative complexity or prolonged surgical time. Furthermore, incomplete documentation of the site of the initial hysterectomy made it impossible to establish whether vault prolapse cases were complications of surgeries performed at our facility.

## Conclusions

POP constituted a substantial proportion of gynecological admissions at MRRH and was the second most frequently encountered PFD after anosphincter injuries. The majority of affected women were treated surgically, with relatively low rates of postoperative complications, while conservative management was rarely utilized. These findings highlight the need for strengthened preventive strategies to reduce the burden of POP and for increased promotion and availability of less invasive management options, particularly vaginal pessaries.
